# Percutaneous Irreversible Electroporation (IRE) of Hepatic Malignancy: A Bi-institutional Analysis of Safety and Outcomes

**DOI:** 10.1007/s00270-018-2120-z

**Published:** 2018-11-21

**Authors:** Sebastian Mafeld, Jen Jou Wong, Nabil Kibriya, Ben Stenberg, Derek Manas, Paul Bassett, Tahira Aslam, Jonathan Evans, Peter Littler

**Affiliations:** 1 Department of Interventional Radiology, Freeman Hospital, Newcastle upon Tyne, NE7 7DN UK; 2 Department of Hepatobiliary Surgery, Freeman Hospital, Newcastle upon Tyne, NE7 7DN UK; 3 Statsconsultancy Ltd, Amersham, Bucks UK; 4 Department of Interventional Radiology, Royal Liverpool University Hospital, Prescot St, Liverpool, L7 8XP UK

**Keywords:** Irreversible electroporation, Hepatic malignancy, Tumour ablation

## Abstract

**Aim:**

Irreversible electroporation (IRE) is a non-thermal ablative option in patients unsuitable for standard thermal ablation, due to its potential to preserve collagenous structures (vessels and ducts) and a reduced susceptibility to heat sink effects. In this series from two large tertiary referral hepatobiliary centres, we aim to assess the safety/outcomes of hepatic IRE.

**Materials and Methods:**

Bi-institutional retrospective, longitudinal follow-up series of IRE for primary hepatic malignancy; [hepatocellular carcinoma (*n* = 20), cholangiocarcinoma (*n* = 3)] and secondary metastatic disease; colorectal (*n* = 28), neuroendocrine (*n* = 1), pancreatic (*n* = 1), breast (*n* = 1), gastrointestinal stromal tumour (GIST, *n* = 1) and malignant thymoma (*n* = 1). Outcome measures included procedural safety/effectiveness, time to progression and time to death.

**Results:**

Between 2013 and 2017, 52 patients underwent percutaneous IRE of 59 liver tumours in 53 sessions. All tumours were deemed unsuitable for thermal ablation. Cases were performed using ultrasound (US) or computed tomography (CT) guidance. A complete ablation was achieved in *n* = 44, (75%) of cases with an overall complication rate of 17% (*n* = 9). Of the complete ablation group, median time to progression was 8 months. At 12 months, 44% were progression-free (95% CI 30–66%). The data suggest that larger lesion size (> 2 cm) is associated with shorter time to progression and there is highly significant difference with faster time to progression in mCRC compared with HCC. Median survival time was 38 months.

**Conclusion:**

This bi-institutional review is the largest UK series of IRE and suggests this ablative technology can be a useful tool, but appears to mainly induce local tumour control rather than cure with HCC having better outcomes than mCRC.

## Introduction

**Fig. 1:**
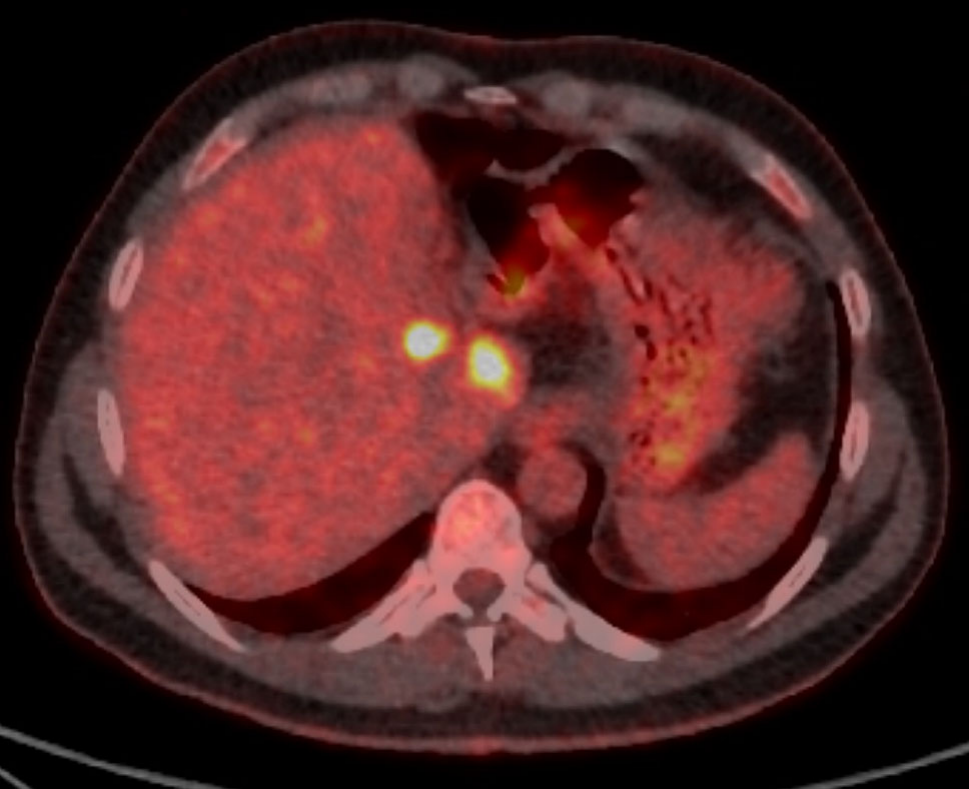
48-year-old male with colorectal liver metastases. Surveillance PET-CT imaging detected recurrence adjacent to the resection margin and either side of the left portal vein unsuitable for thermal ablation

Percutaneous ablation is a treatment option for primary and metastatic hepatic malignancy where surgical resection cannot be performed. Heat-based ablative technologies with radiofrequency (RFA) and microwave (MWA) energy have a growing evidence base to support their safety and effectiveness in the treatment of hepatic malignancy [1, 2]. For tumours located near thermosensitive structures (for example; bile ducts or gallbladder), thermal ablation can induce unwanted injury. Also, for tumour adjacent to large hepatic blood vessels, thermal ablation can have reduced efficacy due to heat sink effects [3]. Irreversible electroporation is a non-thermal ablative technique which can be used in the aforementioned circumstances when thermal ablative techniques may be unsafe or less effective. Unlike thermal ablation which induces cell death through coagulation necrosis, IRE primarily causes cell death through apoptosis [4, 5]. This effect is achieved by placing electrodes in/around a target tumour and applying high voltage electrical currents to induce irreversible nanopore formation in cell membranes which alters cell permeability ultimately leading to apoptosis [6]. A unique feature of IRE is that it does not affect all tissues equally; tissues with higher collagenous tissue content, for example, bile ducts and blood vessels, lack a normal cellular membrane which renders IRE’s ability to induce nanopore formation ineffective, thereby preserving these structures [5]. Therefore, through IRE’s non-thermal mechanism which avoids heat sink and its ability to reduce collateral damage, it has been suggested as an alternative ablative technology in the liver [6]. Pre-clinical studies in animals have confirmed IRE’s ability to induce tumour necrosis while sparing adjacent vulnerable structures and support its safety and effectiveness [7–10]. IRE is commercially available for human treatment as NanoKnife (AngioDynamics, New York, USA). Limited clinical data exist in terms of IRE’s safety and effectiveness in the treatment of hepatic malignancy. The aim of this study is to contribute to the existing literature on local tumour control and complications with the use of IRE in the treatment of both primary and secondary hepatic malignancies (Fig. 1).

## Materials and Methods

A retrospective analysis was performed to identify all patients who had undergone percutaneous hepatic IRE at two large hepatobiliary tertiary referral centres in the UK.

### Patients and Tumour Characteristics

**Fig. 2:**
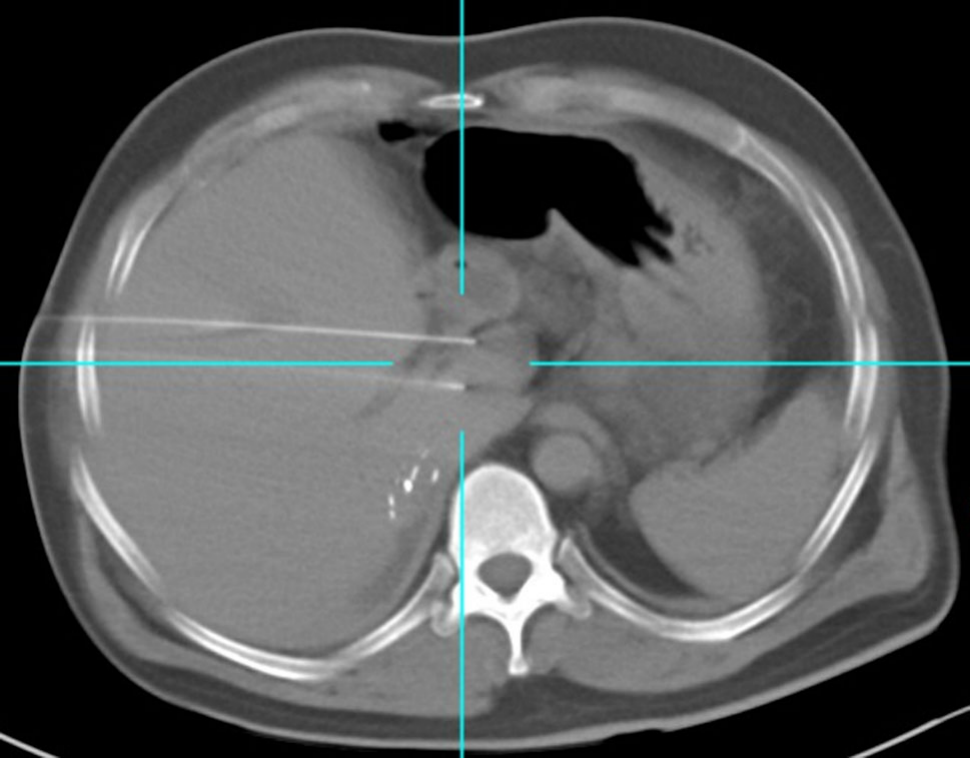
CT demonstrating  parallel IRE electrode position for treatment (third needle not shown).

Between 2013 and 2017, a total of 59 tumours were treated in 52 patients with primary or secondary hepatic malignancy: 43 males and 9 females with a mean age of 64 (range 28–94). All cases were discussed at a multidisciplinary tumour board and were determined to be surgically unresectable and in a location unsuitable for thermal ablation (centrally located in proximity to major vascular structures or adjacent organs). Exclusion criteria for IRE at both institutions included: presence of a cardiac pacemaker, uncontrolled cardiac arrhythmia, or uncorrectable coagulopathy (Fig. 2).

**Fig. 3:**
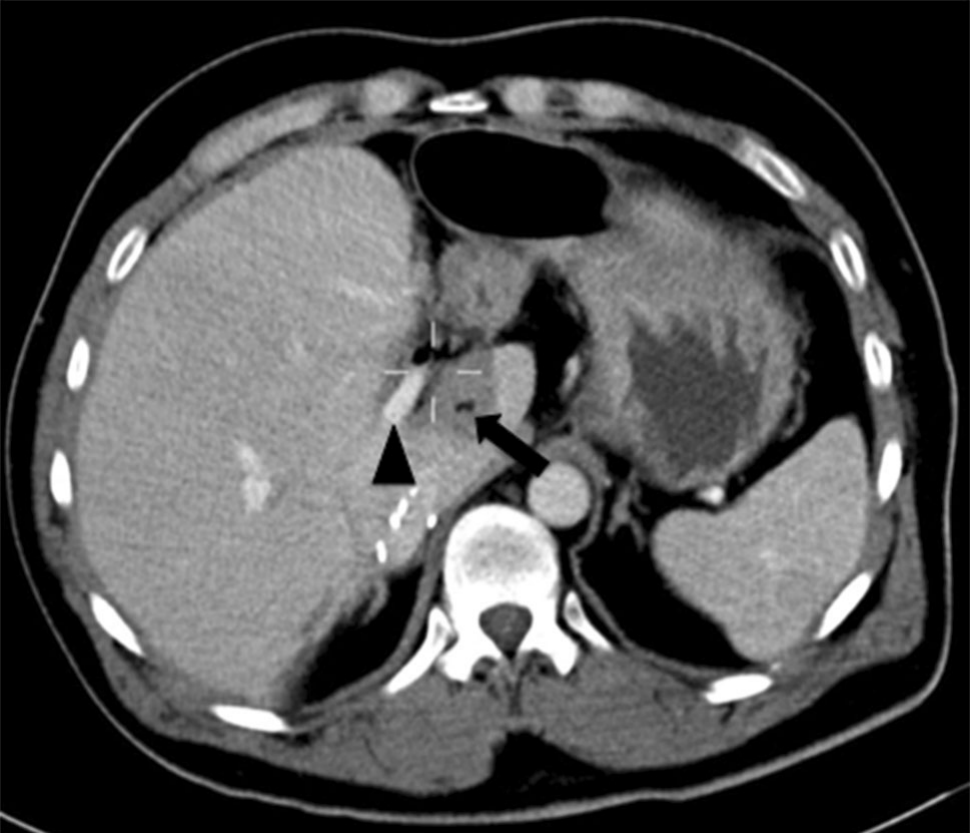
Post-treatment contrast-enhanced portal venous CT scan demonstrates the ablation zone (arrow), surrounding a portal vein branch (arrow head) which remains preserved and patent. The remains alive 8 months post-procedure

Tumour treated included primary hepatic malignancy; hepatocellular carcinoma (*n* = 20), cholangiocarcinoma (*n* = 3) and secondary metastatic disease; colorectal (*n* = 28), neuroendocrine (*n* = 1), pancreatic (*n* = 1), breast (*n* = 1), gastrointestinal stromal tumour (GIST, *n* = 1) and malignant thymoma (*n* = 1). Mean tumour diameter was 2.4 cm (range 0.7–5.2 cm) (Fig. 3).

### Interventional Procedure

All cases were performed using the NanoKnife system in accordance with the manufacturer’s guidance. General anaesthetic with neuromuscular blockade (most commonly using rocuronium bromide) is mandatory to minimise unwanted muscular contraction.

IRE electrodes were percutaneously placed using image guidance as per operator preference. A variety of imaging modalities were employed, the most common being computed tomography guidance (*n* = 35). Other modalities included ultrasound alone (*n* = 13), contrast-enhanced ultrasound (CEUS) in *n* = 8, CT/US fusion (*n* = 2) and combined CT/US in (*n* = 1). Electrodes were placed in a parallel direction with a distance of 1.0–2.0 cm apart. A mean number of electrodes used were 3 (range 2–7) which were sited in order to build an ablation zone encompassing the target lesion and rim of surrounding tissue. Electrode repositioning was carried out as required to enable ablation of the whole lesion. Including initial test pulses, 90 pulses of 1500 v/cm were applied between each electrode pair. Parameters were adjusted if necessary in order to achieve a range of 20–50 Amperes, a level associated with irreversible electroporation and cell death.

The pulses are delivered with ECG gating in the refractory phase after myocardial depolarisation to minimise the risk of cardiac arrhythmias.

Post-intervention patients were transferred to the post-anaesthesia care unit (PACU) for recovery and then transferred back to the ward with a minimum of 6 h bed rest. Mean hospital stay was 3 days (range 1–12).

### Outcome Measures and Statistical Analysis

There were two outcome measures: time to progression and time to death. As not all subjects progressed or died, these were considered as survival outcomes. The outcome was recorded as the time to the progression or death. Those where these outcomes had not occurred were censored to the last time where either progression or death was recorded not to have occurred.

For patients with more than one lesion, progression was deemed to have occurred, if one or more of the lesions progressed. The time to the two outcomes was summarised and displayed graphically using Kaplan–Meier methods. Median survival was calculated, as was survival at specific points in time. Corresponding confidence intervals were calculated for the estimates at specific time points.

Patients were divided into three groups based on their lesion size. For patients with more than one lesion, the largest lesion size was considered. The logrank test was used to compare time to progression and overall survival between groups.

Patients were also divided into groups based on their pathology. The majority of patients had either a CRLM or HCC pathology, and specific comparisons of the outcomes of these two groups only were made using the logrank test. Patients with other pathologies were omitted from these analyses.

## Results

### Adverse Events and Complications

In 53 IRE sessions, 9 (17%) complications occurred in 7 patients (Table 1) and are reported using the CIRSE Quality Assurance Document and Standards for Classification of Complications [11]. Half (*n* = 4) of these complications occurred intra-procedurally with three instances of atrial fibrillation and one subcapsular haematoma. The remaining complications were identified post-operatively; minor pain managed with analgesia (*n* = 2), peritonitis (secondary to gallbladder perforation) and systemic inflammatory response (SIRS) syndrome leading to death. The death was observed 9 days post-IRE (mortality 1.8%) in which a patient with pre-existing common bile duct stones developed cholangitis, branch portal vein occlusion and developed SIRS, but died despite intensive care support.

**Table 1:** All complications during and after 53 IRE procedures. Categorised by CIRSE classification system

Classification (CIRSE)	Event	Number
1	Atrial fibrillation	3
	Minor pain	2
2		0
3	Subcapsular haematoma	1
4	Gallbladder perforation with resultant bile leak and peritonitis	1
	Systemic inflammatory response syndrome	1
5		0
6	Death	1

### Outcomes, Tumour Response and Survival

Technical success was defined as a complete response on first follow-up imaging using either contrast-enhanced CT or MRI at 4–8 weeks post-ablation. A complete ablation was achieved in *n* = 44, (75%) of ablations in 37 patients. All radiology images were reviewed by radiologists with experience in post-ablation and hepatobiliary imaging. For HCC, a complete response was reported according to mRECSIST. An incomplete ablation was observed in *n* = 13 cases (22%), one patient was lost to follow-up, and 1 patient died as outlined above. Where patients were found to have an incomplete ablation, a second attempt at IRE was not performed, and instead, patients were managed by non-interventional treatments.

**Fig. 4:**
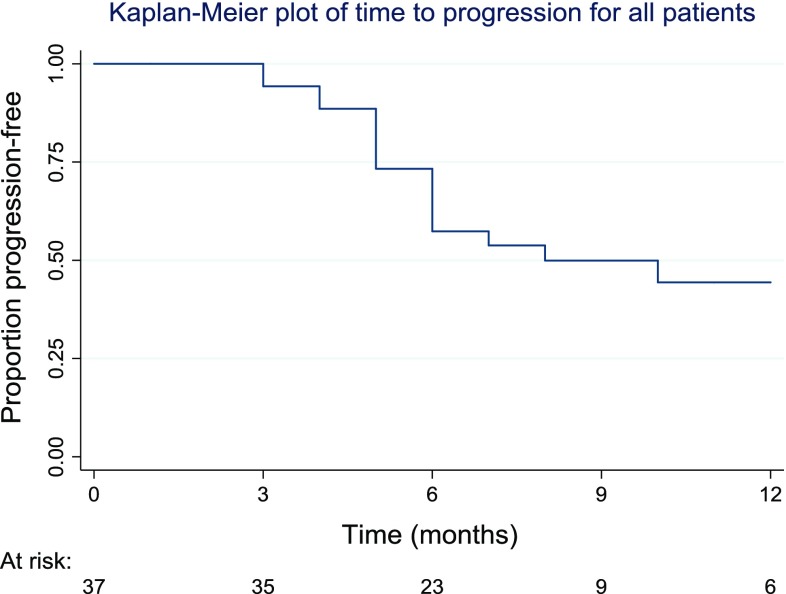
A graphical illustration of the time to progression is shown in the subsequent Kaplan–Meier plot. The plot is capped at 12 months, as follow-up beyond this time occurred in only a smaller number of patients

Of the complete ablation patient group, imaging follow-up was planned to continue at three-monthly intervals after the initial post-ablation imaging at 4–8 weeks. Median time to progression was observed to be 8 months. At 12 months, the percentage that was progression-free was 44% (95% CI 26–62%) (Fig. 4).

**Fig. 5:**
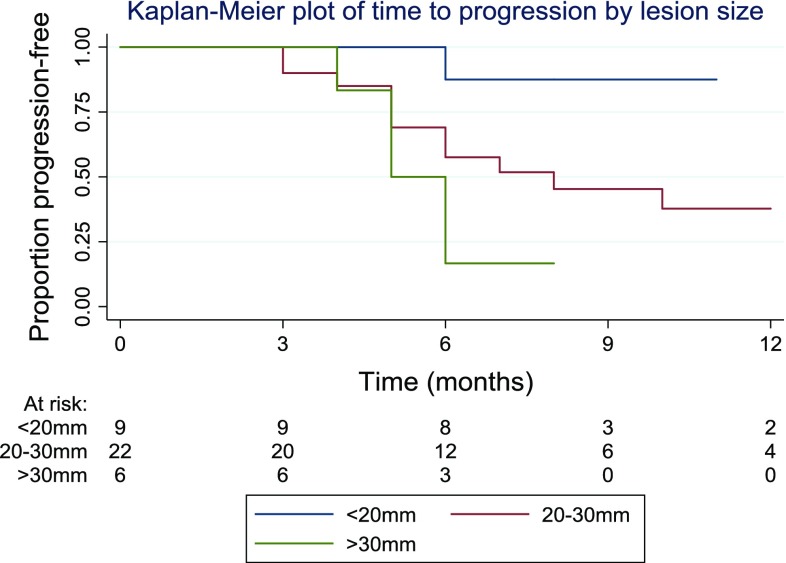
A graphical illustration of the time to progression in the different groups based on lesion size

Patients were divided into three categories based on their lesion size. 9 patients (24%) had lesion of under 20 mm, 22 patients (59%) had a lesion of 20–30 mm, whilst 6 patients (16%) had a lesion of over 30 mm. A graphical illustration of the time to progression in the different groups is shown in Fig. 5. The logrank test was used to compare the progression times in the three groups. The results suggested evidence of a difference between groups (*p* = 0.04) with larger lesion size associated with a shorter time to progression.

**Fig. 6:**
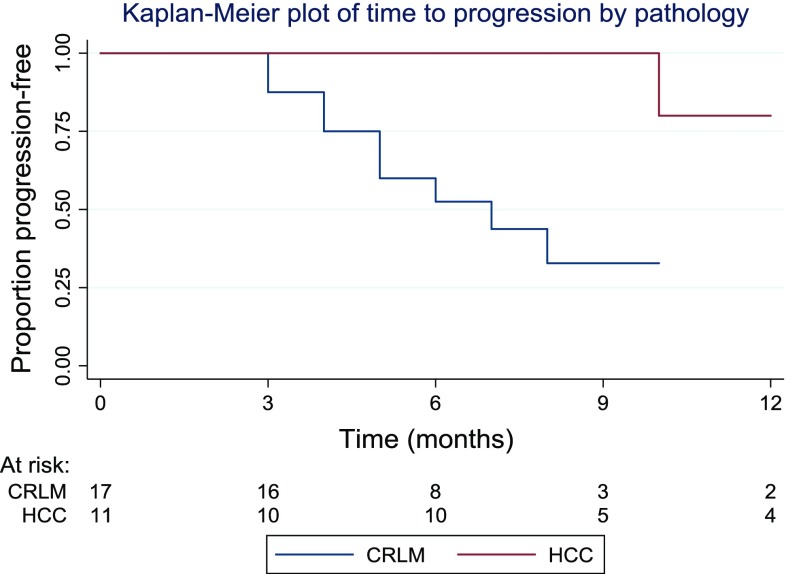
Highly significant difference in time to progression in the two pathologies (*p* = 0.004)

Comparisons were also made between the two most common pathologies: CRLM and HCC. The data suggested that 17 patients (46% of all patients) were in the CRLM category, whilst 11 were HCC patients (30%). The logrank test suggested a highly significant difference in time to progression in the two pathologies (*p* = 0.004). The Kaplan–Meier plot (Fig. 6) shows that progression was faster in the CRLM group than in HCC patients.

**Fig. 7:**
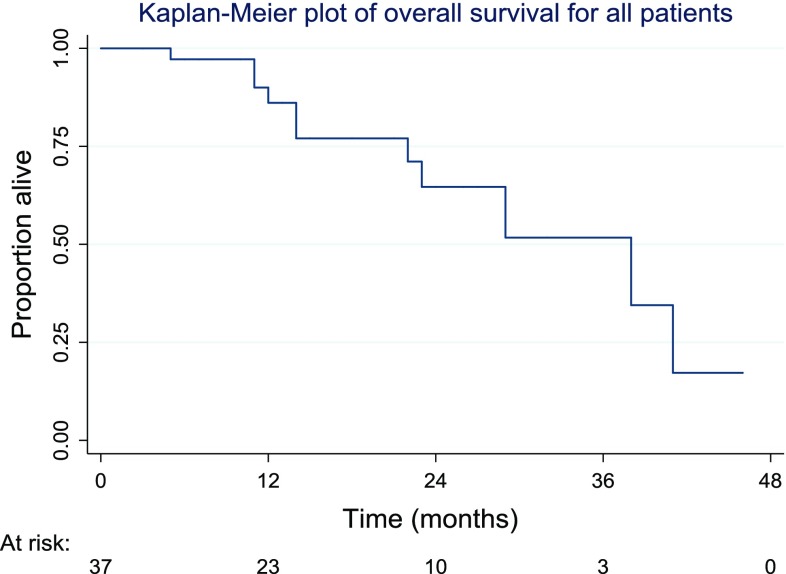
Kaplan–Meier survival curse for group as a whole

**Fig. 8:**
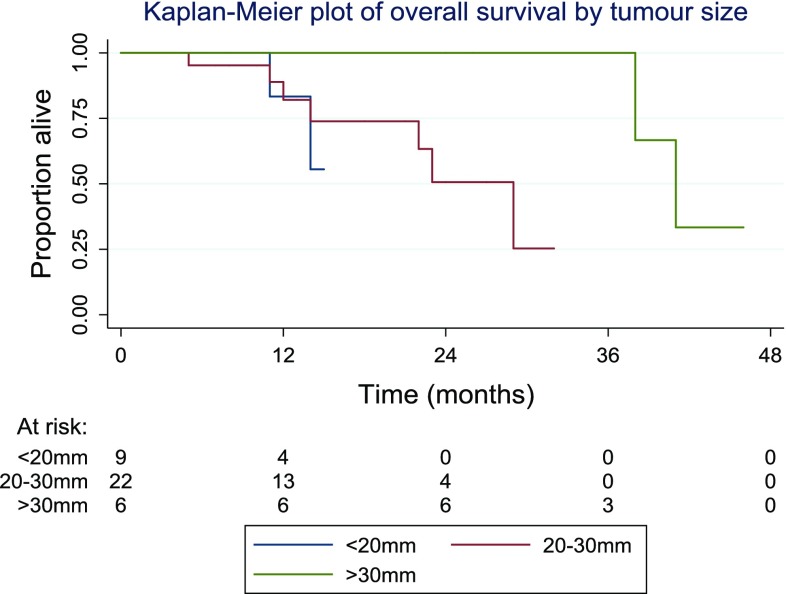
Survival in the different subgroups subdivided by tumour size

The second outcome examined was patient survival, the time to death. The median survival time was found to be 38 months, with an inter-quartile range from 22 to 41 months. Patient survival at 12 months was 90% (95% CI 72–97%), at 24 months was 65% (95% CI 40–81%) and at 36 months was 52% (95% CI 22–75%). The Kaplan–Meier plot (Fig. 7) demonstrates the survival times in the patient group as a whole. While Fig. 8 is subdivided the patients by lesion size and the logrank test suggested slight evidence of a difference in overall survival between groups, this difference was not statistically significant (*p* = 0.06). These results are counter-intuitive, with the longest survival in the group with the largest lesions, but limited conclusions can be drawn from this due to small sample size in this subgroup analysis.

## Discussion

In patients with resectable hepatic malignancy, surgical resection is regarded as the gold standard of care. However, data from two of the most common hepatic cancers: metastatic colorectal cancer (mCRC) and hepatocellular carcinoma (HCC), suggest that less than 30% of patients are typically suitable for surgery. Thermal ablation with RFA and MWA has a proven role in the management of unresectable hepatic malignancy [12, 13]. In small lesions, ablation can rival surgical resection in terms of local tumour control [12]. Ablation is also associated with a low morbidity and mortality. In a meta-analysis of over 15,000 patients, the morbidity (major complication rate defined as any symptom that developed after ablation and persisted for more than 1 week, or those that delayed hospital discharge, threatened the patient’s life or led to substantial morbidity and disability) was 4.1% for RFA and 4.6% for MWA [14, 15]. Mortality was calculated at 0.15% for RFA and 0.23% for MWA. Not all hepatic lesions are suitable for thermal ablation due to the danger of damaging adjacent thermosensitive structures such as the gallbladder and central bile ducts. Furthermore, ablation adjacent to large vessels can be ineffective due to heat sink effects or result in vessel thrombosis [16]. IRE is a comparatively novel non-thermal ablation technology which can induce tumour necrosis while sparing adjacent vulnerable structures. Emerging small data series support IRE’s safety and effectiveness in the liver [7–10].

A recent systematic review has suggested a complication rate for hepatic IRE ranging from 11 to 36% [17]. The data from our series are at the lower end of this rate at 17%. However, these figures are considerably higher than thermal ablation complication rates. This increase may be related to the fact IRE requires a minimum of two electrodes and ablations are more frequently adjacent to vital hepatic structures. Of note, the cardiac arrhythmias appear more frequent with IRE, while the vicinity of electrodes close to the heart has been suggested as a possible cause, but the precise mechanism is not fully understood. It has also been shown that subclinical myocardial injury can occur with IRE as evidenced by elevations in high-sensitive troponin I [18]. Cardiac arrhythmias rarely impair completion of an IRE procedure, as was the case in our three instances of atrial fibrillation which were all either self-limiting or medically managed [19]. Ablation of large tumour volumes (40% of liver) has been shown in an animal model to induce alterations in serum potassium levels due to ionic shifts with nanopore formation resulting in the potential for electrocardiogram arrhythmias, but this mechanism is not felt to be applicable in this study as lesional volume was considerably smaller [20].

A systematic review of mainly single-centre retrospective reviews has shown hepatic IRE to have a primary efficacy of 67–100% [17]. Published data from multicentre prospective trials in hepatic IRE are still awaited, but preliminary presented results from (clinicaltrials.gov ID:NCT01078415) for biopsy proven early-stage HCC have indicated a 1-month complete response rate of 77% [21]. Data from our study have indicated a 75% complete response rate at the first follow-up imaging, which is within the range of the aforementioned published studies. One challenging factor which is rarely mentioned in the literature is the difficulty in interpretation of post-IRE imaging which may impact on the primary efficacy outcomes. Imaging appearances post-IRE remain at an investigative stage; therefore, reported efficacy outcomes should be interpreted with caution [22, 23]. Small histopathologic studies have suggested that imaging responses to IRE may be an inaccurate reflection of the ablation zone [24, 25].

This study and the literature as a whole on hepatic IRE currently suggest that IRE is not as effective as its thermal ablation counterparts, which are able to rival surgical resection [26]. Caution should be observed in case selection with IRE as we have shown statistically significant differences in outcomes with both size and pathology with small (< 2 cm) HCCs having the best outcomes in terms of primary efficacy and longer-term tumour control. Other studies have indicated tumour volume of > 5 cm^3^ and underlying disease type (HCC, cholangiocellular carcinoma or metastatic disease) as independent risk factors for early local recurrence [27].

The reasons for differences between IRE and thermal ablation in terms of primary efficacy and local recurrence are uncertain. The technical challenges of placing multiple electrodes in parallel orientation into challenging hepatic locations may be a factor, and a learning curve of at least five cases has been suggested [28]. Traditional teaching with thermal ablation suggests a 1-cm circumferential ablation zone around a lesion to achieve recurrence-free survival results rivalling surgery [26]. With IRE, electrodes are typically placed at the periphery of a lesion with the electroporation effect extending up to 5 mm beyond the electrode position, and this means the margins achieved using current IRE ablation protocols are not equivalent to thermal ablation. Further investigation into optimum ablation protocols beyond mathematical models is needed [29].

The retrospective nature, relatively small sample size and heterogeneous group of patients with varying pathologies represent the largest limitation to this review. Further heterogeneity exists with the imaging follow-up protocol which employs both CT and MRI at varied time points post-procedure. All patients were, however, planned to have imaging follow-up at 4–8 weeks post-treatment. As the current study was not designed as a trial, ultimately, the information presented reflects ‘real-world data’ from two large hepatobiliary centres. The nature of IRE is such that only a limited patient cohort is suitable for this type of ablation, and it therefore unlikely a gold standard randomised control trial would be possible for this ablation technology. The data obtained for the two largest groups: HCC and CRLM add to the existing literature and should therefore be of use in the process of clinical decision-making. No definite conclusions can be drawn regarding other metastatic malignancies.

## Conclusion

This bi-institutional study represents the largest follow-up series to date in the UK regarding hepatic IRE. Our data suggest that lesion size < 2 cm and HCC represent the ‘optimum’ case selection for IRE. However, even within this group results do not equal the response rates published for thermal ablation. IRE may therefore be an attractive ablation option in patients with no other treatment option, but interventional radiologists should remain aware of the uncertainties regarding this technology.

## References

[1] Pathak S, Jones R, Tang JMF, Parmar C, Fenwick S, Malik H (2011). Ablative therapies for colorectal liver metastases: a systematic review. Colorectal Dis Off J Assoc Coloproctol G B Irel..

[2] Chinnaratha MA, Chuang MA, Fraser RJL, Woodman RJ, Wigg AJ (2016). Percutaneous thermal ablation for primary hepatocellular carcinoma: a systematic review and meta-analysis. J Gastroenterol Hepatol.

[3] Narayanan G, Froud T, Suthar R, Barbery K (2013). Irreversible electroporation of hepatic malignancy. Semin Interv Radiol.

[4] Poulou LS, Botsa E, Thanou I, Ziakas PD, Thanos L (2015). Percutaneous microwave ablation vs radiofrequency ablation in the treatment of hepatocellular carcinoma. World J Hepatol.

[5] Jourabchi N, Beroukhim K, Tafti BA, Kee ST, Lee EW (2014). Irreversible electroporation (NanoKnife) in cancer treatment. Gastrointest Interv.

[6] Savic LJ, Chapiro J, Hamm B, Gebauer B, Collettini F (2016). Irreversible electroporation in interventional oncology: where we stand and where we go. ROFO Fortschr Geb Rontgenstr Nuklearmed.

[7] Sánchez-Velázquez P, Castellví Q, Villanueva A, Iglesias M, Quesada R, Pañella C (2017). Long-term effectiveness of irreversible electroporation in a murine model of colorectal liver metastasis. Sci Rep.

[8] Miller L, Leor J, Rubinsky B (2005). Cancer cells ablation with irreversible electroporation. Technol Cancer Res Treat.

[9] Charpentier KP, Wolf F, Noble L, Winn B, Resnick M, Dupuy DE (2011). Irreversible electroporation of the liver and liver hilum in swine. HPB.

[10] Guo Y, Zhang Y, Klein R, Nijm GM, Sahakian AV, Omary RA (2010). Irreversible electroporation therapy in the liver: longitudinal efficacy studies in a rat model of hepatocellular carcinoma. Cancer Res.

[11] Filippiadis DK, Binkert C, Pellerin O, Hoffmann RT, Krajina A, Pereira PL (2017). Cirse quality assurance document and standards for classification of complications: the cirse classification system. Cardiovasc Intervent Radiol.

[12] Tanis E, Nordlinger B, Mauer M, Sorbye H, van Coevorden F, Gruenberger T (2014). Local recurrence rates after radiofrequency ablation or resection of colorectal liver metastases. Analysis of the european organisation for research and treatment of cancer #40004 and #40983. Eur J Cancer.

[13] Verslype C, Van Cutsem E, Dicato M, Arber N, Berlin JD, Cunningham D (2009). The management of hepatocellular carcinoma. Current expert opinion and recommendations derived from the 10th world congress on gastrointestinal cancer, Barcelona, 2008. Ann Oncol Off J Eur Soc Med Oncol.

[14] Goldberg SN, Charboneau JW, Dodd GD, Dupuy DE, Gervais DA, Gillams AR (2003). Image-guided tumor ablation: proposal for standardization of terms and reporting criteria. Radiology.

[15] Lahat E, Eshkenazy R, Zendel A, Zakai BB, Maor M, Dreznik Y (2014). Complications after percutaneous ablation of liver tumors: a systematic review. Hepatobiliary Surg Nutr.

[16] Chiang J, Hynes K, Brace CL. Flow-dependent vascular heat transfer during microwave thermal ablation. In: Annual international conference of the IEEE engineering in medicine and biology society, Aug 2012. 2012, p. 5582–5. 10.1109/EMBC.2012.6347259 PMC3563104 23367194

[17] Scheffer HJ, Nielsen K, de Jong MC, van Tilborg AAJM, Vieveen JM, Bouwman ARA (2014). Irreversible electroporation for nonthermal tumor ablation in the clinical setting: a systematic review of safety and efficacy. J Vasc Interv Radiol JVIR.

[18] Kostrzewa M, Tueluemen E, Rudic B, Rathmann N, Akin I, Henzler T (2018). Cardiac impact of R-wave triggered irreversible electroporation therapy. Heart Rhythm.

[19] Kambakamba P, Bonvini JM, Glenck M, Castrezana López L, Pfammatter T, Clavien P-A (2016). Intraoperative adverse events during irreversible electroporation-a call for caution. Am J Surg.

[20] Sánchez-Velázquez P, Castellví Q, Villanueva A, Quesada R, Pañella C, Cáceres M (2016). Irreversible electroporation of the liver: Is there a safe limit to the ablation volume?. Sci Rep.

[21] Lencioni R, Izzo F, Crocetti L, Vilgrain V, Abdel-Rehim M, Bianchi L (2012). Abstract No LB12: a prospective, multicenter phase II clinical trial using irreversible electroporation for the treatment of early stage HCC. J Vasc Interv Radiol.

[22] Felker ER, Dregely I, Chung DJ, Sung K, Osuagwu FC, Lassman C (2017). Irreversible electroporation: defining the MRI appearance of the ablation zone with histopathologic correlation in a Porcine liver model. Am J Roentgenol.

[23] Padia SA, Johnson GE, Yeung RS, Park JO, Hippe DS, Kogut MJ (2016). Irreversible electroporation in patients with hepatocellular carcinoma: immediate versus delayed findings at MR imaging. Radiology.

[24] Abdelsalam ME, Chetta JA, Harmoush S, Ensor J, Javadi S, Dixon K (2013). CT findings after irreversible electroporation ablation in a porcine model: radiologic-pathologic correlation. J Vasc Interv Radiol.

[25] Gonzalez-Beicos A, Venkat S, Songrug T, Poveda J, Garcia-Buitrago M, Poozhikunnath Mohan P (2015). Irreversible electroporation of hepatic and pancreatic malignancies: radiologic-pathologic correlation. Tech Vasc Interv Radiol.

[26] McDermott S, Gervais DA (2013). Radiofrequency ablation of liver tumors. Semin Interv Radiol.

[27] Niessen C, Igl J, Pregler B, Beyer L, Noeva E, Dollinger M (2015). Factors associated with short-term local recurrence of liver cancer after percutaneous ablation using irreversible electroporation: a prospective single-center study. J Vasc Interv Radiol.

[28] Philips P, Hays D, Martin RCG (2013). Irreversible electroporation ablation (IRE) of unresectable soft tissue tumors: learning curve evaluation in the first 150 patients treated. PLoS ONE.

[29] Edd JF, Davalos RV (2007). Mathematical modeling of irreversible electroporation for treatment planning. Technol Cancer Res Treat.

